# Case Report: Different faces of LRBA deficiency in five Moroccan families

**DOI:** 10.3389/fimmu.2026.1807421

**Published:** 2026-05-01

**Authors:** Mohamed Hbibi, Ahamada Elamine, Ibtihal Benhsaien, Mounia Lakhdar Idrissi, Samira Tizki, Jalila El Bakkouri, Moustapha Hida, Fatima Ailal, Ahmed Aziz Bousfiha

**Affiliations:** 1 Department of Pediatric Hematology-Oncology, SHOP, CHU Hassan II, Fez, Morocco; 2 Laboratory of Clinical Immunology, Infection and Autoimmunity (LICIA). Faculty of Medicine and Pharmacy. Hassan II University., Casablanca, Morocco; 3 Department of Pediatric Infectious Diseases and Clinical Immunology, Mother-Child Hospital Abderrahim El Harouchi, Ibn Rochd University Hospital Center., Casablanca, Morocco; 4 Department of Pediatrics, CHU Hassan II, Fez, Morocco; 5 Pediatrics department, Faculty of Medicine and Pharmacy. University Hospital Center Mohammed VI, Ibn ZOHR University, Agadir, Morocco

**Keywords:** Autoimmune cytopenias, immune dysregulation, inborn error of immunity, LRBA deficiency, LYMPHOPROLIFERATION

## Abstract

LPS-responsive beige-like anchor protein (LRBA) deficiency is a primary immunodeficiency belonging to the spectrum of common variable immunodeficiency disorders, frequently associated with immune dysregulation, autoimmunity, and lymphoproliferation. Its clinical presentation is highly heterogeneous, ranging from isolated autoimmune cytopenias to severe multisystem involvement. Here, we report five cases of LRBA deficiency from five Moroccan families, highlighting the broad clinical variability of this condition. The first patient presented with chronic immune thrombocytopenic purpura (ITP) as the initial and predominant manifestation, associated with hypogammaglobulinemia. The second patient developed ITP complicated by a hemorrhagic syndrome and significant lymphoproliferation, including splenomegaly and hepatomegaly. The third patient initially presented with autoimmune hemolytic anemia (AIHA), followed by splenomegaly. The fourth patient exhibited a complex clinical course, beginning with bicytopenia and progressing to enteropathy, arthritis, pneumopathy, and type 1 diabetes mellitus, associated with splenomegaly, hepatomegaly, and lymphadenopathy. Finally, the fifth patient initially presented with ITP and subsequently developed bilateral panuveitis, arthritis, and recurrent respiratory infections. Targeted next-generation sequencing identified homozygous pathogenic variants in the *LRBA* gene in all five patients, consistent with a loss-of-function mechanism. These included the nonsense variants c.3811C>T (p.Arg1271*) and c.5692G>T (p.Glu1898*), a large deletion encompassing exons 30 to 34, the frameshift variant c.5060_5067delACATACCA (p.Asn1687Serfs*21), and an extended deletion involving part of exon 4 (c.476_549 + 580del). All patients required immunosuppressive therapy and/or immunoglobulin replacement. LRBA deficiency should be considered in children presenting with autoimmune cytopenias, lymphoproliferation, and multisystem autoimmunity. The marked heterogeneity of clinical manifestations underscores the importance of early molecular diagnosis to guide therapeutic decision-making. In our experience, prompt recognition and the initiation of targeted therapies, such as abatacept or early hematopoietic stem cell transplantation, may improve patient outcomes.

## Introduction

LRBA (*Lipopolysaccharide-Responsive Beige-like Anchor*) deficiency is an inborn error of immunity (IEI) caused by biallelic mutations in the *LRBA* gene, most often resulting in absent or markedly reduced expression of the LRBA protein (1). The LRBA protein belongs to the BEACH protein family, which is involved in the regulation of intracellular vesicular trafficking and exocytosis (2). LRBA interacts with CTLA-4 through its BEACH-WD domain, ensuring the maintenance of intracellular CTLA-4 pools and its rapid mobilization to the surface of regulatory T lymphocytes (Tregs) (1, 3). CTLA-4 plays a central role in immune regulation by removing the costimulatory molecules CD80/CD86 from antigen-presenting cells through transendocytosis (4). In the context of LRBA deficiency, CTLA-4–containing vesicles are misdirected to lysosomes and subsequently degraded, leading to reduced surface expression of CTLA-4 and impaired suppressive function of Tregs (5).

Initially, LRBA deficiency was described as a form of common variable immunodeficiency (CVID) complicated by autoimmune manifestations. Indeed, affected patients frequently presented with recurrent infections, hypogammaglobulinemia, as well as quantitative and functional abnormalities of B-cell subsets (6). However, the accumulation of clinical and immunological data has allowed a more precise characterization of the pathophysiological spectrum of the disease. According to the current classification of the International Union of Immunological Societies (IUIS), LRBA deficiency is now categorized among immune dysregulation disorders with autoimmunity (7), mainly due to Treg dysfunction, which plays a central role in the breakdown of immune tolerance.

LRBA deficiency was first described in 2012 in five children carrying four distinct homozygous mutations in the *LRBA* gene, resulting in complete loss of protein expression (8). The disease typically manifests in early childhood and is characterized by a broad clinical spectrum, including autoimmune manifestations, enteropathy, and organomegaly, as well as hypogammaglobulinemia, recurrent respiratory infections, polyendocrinopathy, growth retardation, and neurological and skeletal involvement (1, 9). Immunological abnormalities reported in patients with LRBA deficiency are diverse and affect both cellular and humoral immunity. These include impaired activation and proliferation of T lymphocytes, as well as an increased frequency of circulating follicular helper T cells (10). With respect to humoral immunity, defects in antigen-specific antibody responses have been reported, together with decreased production of IgG immunoglobulins. In addition, intrinsic B-cell abnormalities have been identified, characterized by reduced autophagy and increased apoptosis (6). Consequently, most patients with LRBA deficiency exhibit a reduction in switched memory B cells and plasmablasts (6, 11).

To date, slightly more than two hundred patients with LRBA deficiency have been reported worldwide, confirming the rarity of this autosomal recessive inherited disorder (12). Since its initial identification, more than one hundred pathogenic variants in the *LRBA* gene have been described in the literature (13). Here, we present the clinical, immunological, and genetic characteristics of five patients from five Moroccan families with LRBA deficiency, highlighting the heterogeneity and diversity of the clinical manifestations associated with this condition.

## Methods

### Patients and clinical investigations

This study includes five patients with LRBA deficiency from five unrelated families, who were managed, diagnosed, and followed at the Department of Pediatric Infectious Diseases and Clinical Immunology of the Abderrahim El Harouchi Mother and Child Hospital, Ibn Rochd University Hospital Center, Casablanca, Morocco. A comprehensive clinical evaluation was performed for each patient, including detailed medical history, physical examination, and appropriate laboratory investigations. Family pedigrees were established based on direct interviews conducted with the parents.

### Immunophenotypic analysis by flow cytometry

Lymphocyte subset analysis was performed by flow cytometry according to standardized protocols. Peripheral whole blood samples were collected in EDTA anticoagulant tubes and analyzed within a maximum of 24 hours after collection. Following red blood cell lysis using an ammonium chloride–based lysis buffer provided by BD Biosciences, leukocytes were incubated with a panel of fluorochrome-conjugated monoclonal antibodies, including anti-CD45-V500-PerCP-Cy5.5, anti-CD3-FITC, anti-CD4-PE-Cy7, anti-CD8-APC-Cy7, anti-CD19-APC, and anti-CD16 + 56-PE (BD Biosciences). Data acquisition was performed on a FACSLyric™ flow cytometer (BD Biosciences). Results were interpreted using age-adjusted reference values.

### Genetic analysis

Genomic DNA was extracted from peripheral blood leukocytes of the patients using standard protocols and the Invitrogen kit (Thermo Fisher Scientific), reference K182001, lot number 2781570. Targeted next-generation sequencing (NGS) was performed using a customized gene panel covering the main genes involved in primary immunodeficiencies, including LRBA. Sequencing libraries were prepared by hybrid capture and sequenced on an Illumina platform. The obtained reads were aligned to the human reference genome (GRCh37). Variant calling, annotation, and nomenclature were performed according to the recommendations of the Human Genome Variation Society (HGVS).

### Ethical considerations

This study was conducted in accordance with the ethical principles outlined in the Declaration of Helsinki. Written informed consent was obtained from the legal guardians of the patients for clinical investigations, genetic analyses, and the publication of anonymized clinical data. The study protocol was approved by the Ethics Committee of the Faculty of Medicine and Pharmacy, Hassan II University of Casablanca (approval number: 06/2023), to which the Ibn Rochd University Hospital Center, Casablanca, Morocco, is affiliated.

## Results

### Clinical presentation

#### Family 1 (case 1)

**Figure 1:**
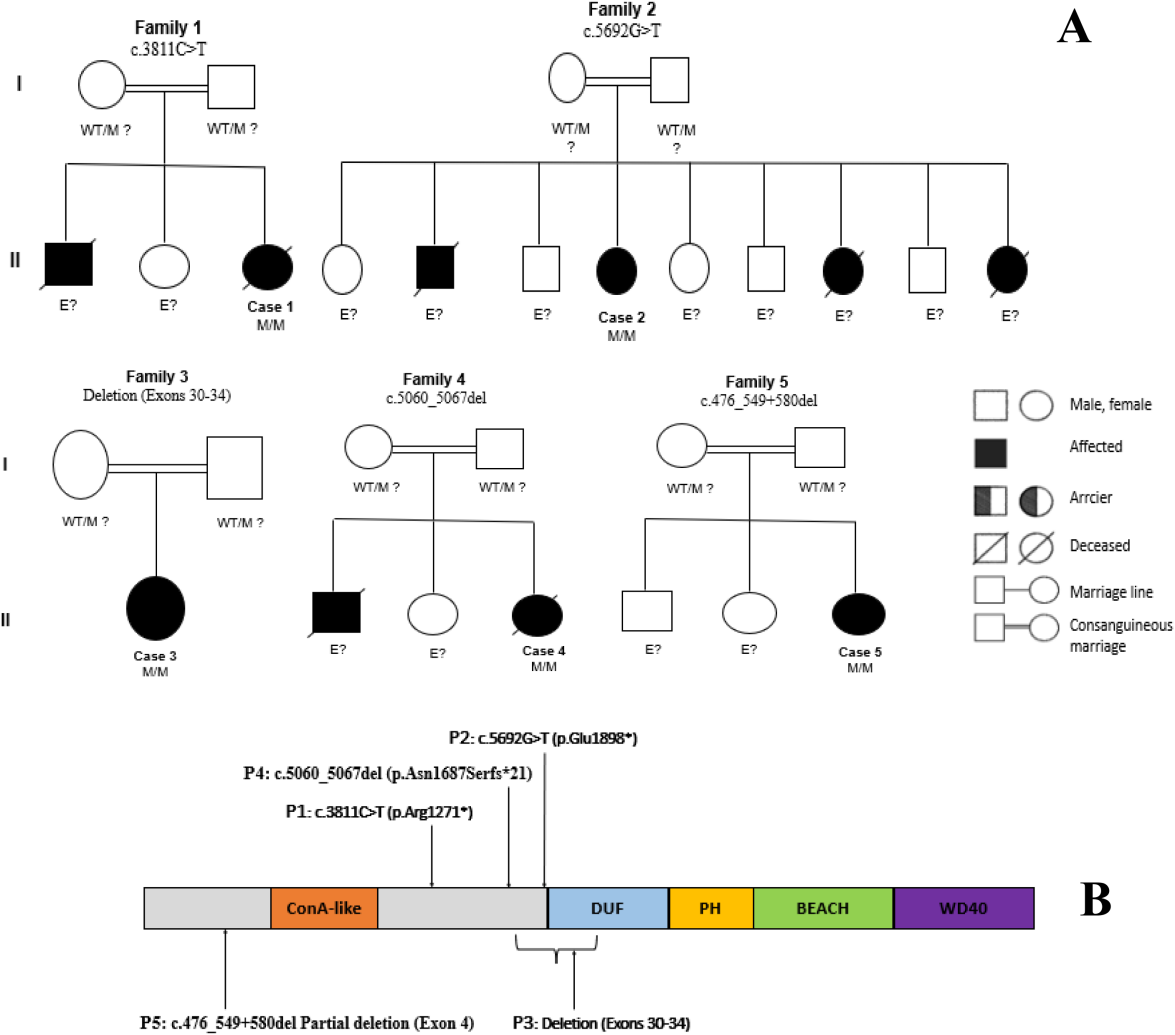
**(A)** Pedigrees of five unrelated Moroccan families with autosomal recessive LRBA deficiency. All patients were born to consanguineous parents. Affected individuals (black-filled symbols) are homozygous for distinct mutations in the LRBA gene. A diagonally slashed square indicates a deceased brother, and a diagonally slashed circle indicates a deceased sister, neither of whom underwent genetic testing. Parents are presumed heterozygous carriers (WT/M)?. Individuals labeled E? have unknown genetic status. WT, wild-type allele; M, mutant allele. Squares represent males; circles represent females. Double horizontal lines indicate consanguineous unions. **(B)** An approximate schematic representation of domains and location of mutations in LRBA at the protein level. BEACH domain of Beige and Chediak-Higashi, ConA-like concanavalin A-like lectin/glucanase domain, DUF domain of unknown function, PH Pleckstrin homology domain, adapted from Alkhairy et al. (14).

Patient 1 is a 15-year-old girl born from a second-degree consanguineous marriage (**Figure 1A**) who was followed for hypogammaglobulinemia associated with Evans syndrome, including immune thrombocytopenic purpura and autoimmune hemolytic anemia. Her medical history included severe growth retardation (–4 SD), mild cognitive delay, Langerhans cell histiocytosis treated orthopedically at 4 months of age, and tonsillectomy at 3 years. The onset of symptoms occurred at 11 years with recurrent cutaneous purpura and mucosal bleeding episodes. After extensive investigations, a diagnosis of immune thrombocytopenic purpura was established, followed by the development of autoimmune hemolytic anemia, constituting Evans syndrome.

Laboratory evaluation revealed severe anemia with hemoglobin of 6.6 g/dL, leukocytosis of 21,690/mm³ with neutrophilic predominance (17,430/mm³), profound thrombocytopenia (5,000/mm³), and lymphopenia (980/mm³). Bone marrow examination showed peripheral thrombocytopenia without significant marrow abnormalities. Lymphocyte immunophenotyping demonstrated elevated CD3 (3,112/mm^3^), CD4 (1,530/mm^3^), CD8 (1,432/mm^3^), reduced CD19 (305/mm^3^), and NK cells counts were normal (77/mm^3^). Serum immunoglobulin quantification revealed an isolated hypogammaglobulinemia, with decreased IgG levels (4.88 g/L) and IgA (0.43 g/L), while IgM (0.58 g/L), and IgE (54, 23) were within the normal reference ranges (**Table 1**). The patient received multiple lines of therapy including systemic corticosteroids, methylprednisolone pulses, and intravenous immunoglobulin infusions during acute bleeding episodes, followed by monthly IVIG maintenance. Despite these interventions, the patient died from severe hemorrhagic syndrome associated with impaired consciousness.

**Table 1:** Laboratory and immunological findings of five Moroccan patients with LRBA deficiency.

Paramètre	Case 1/Reference ranges	Case 2/Reference ranges	Case 3/Reference ranges	Case 4/Reference ranges	Case 5/Reference ranges
WBC (/mm³)	21 690 (4000-9200) ↑	4080 (5600-12200) ↓	2010 (5000–10100) ↓	1980 (4000-9200) ↓	14 300 (4000-9200) ↑
Neutrophils (/mm³)	17 430 (1620-6460) ↑	890 (1410–5840) ↓	310 (1410–5840) ↓	1100 (1410–5840) ↓	12 870 (1620-6460) ↑
Lymphocytes (/mm³)	980 (1,17-3,05) ↓	1013 (2130–7050) ↓	1150 (2130–7050) ↓	770 (1410-3650) ↓	715 (1,17-3,05) ↓
Hemoglobin (g/dL)	6,6 (11,8-14,8) ↓	5,9 (10.6–13.5) ↓	9,8 (10.6–13.5) ↓	5,8 (11,7-14,0) ↓	9,9 (11,8-14,8) ↓
Platelets (/mm³)	5 000 (190000-388000) ↓	4000 (222000–444000) ↓	141 000 (222000–444000)	180 000 (222000–444000)	126 000 (190000-388000) ↓
IgG (g/L)	4,88 (6,36–16,10) ↓	4,88 (4,86–19,70)	5,97 (4,57–11,20)	7,54 (4,57–11,20)	0;00 (6,88–24,30) ↓
IgA (g/L)	0,43 (0,56–3,05) ↓	0,68 (0,67–1,35)	0,48 (0,36–1,92)	0,42 (0,36–1,92)	0,24 (0,46–3,85) ↓
IgM (g/L)	0,58 (0,42–1,97)	0,95 (0,43–2,36)	0,61 (0,58–1,98)	1,01 (0,58–1,98)	0,1 (0,61–3,23) ↓
IgE (IU/mL)	54,23	13,90	50	26,7	52
CD3^+^ T cells(/mm³)	3112 (1000-2200) ↑	1569 (2100-6200) ↓	417 (1400–3700) ↓	2213 (1400-3700)	1125 (1000-2200)
CD4^+^ T cells (/mm³)	1530 (530-1300) ↑	697 (1300-3400) ↓	206 (700–2200) ↓	325 (700–2200) ↓	455 (530-1300) ↓
CD8^+^ T cells (/mm³)	1432 (330-920) ↑	717 (620-2000)	183 (490–1300) ↓	503 (490–1300)	638 (330-920)
CD19^+^ B cells (/mm³)	305 (110-570)	58 (720-2600) ↓	190 (390–1400) ↓	234 (390–1400) ↓	0 (110-570) ↓
NK (CD16^+^/56^+^) cells (/mm³)	77 (70-480)	252(180-920)	63 (130–720) ↓	103 (130–720) ↓	89 (70-480)
LRBA variant	c.3811C>T (p.Arg1271*)	c.5692G>T (p.Glu1898*)	Deletion (Exons 30-34)	c.5060_5067del (p.Asn1687Serfs*21)	c.476_549 + 580del Partial deletion (Exon 4)
ACMG classification	Pathogène homozygote (PVS1, PM2, PP3, PP5)	Pathogène homozygote (PVS1, PM2, PP5)	Pathogène homozygote (PVS1, PM2, PS4)	Pathogène homozygote (PVS1, PM2, PP5)	Likely Pathogenic homozygote (PVS1, PM2)

*Reference ranges: hematology values were adapted from CALIPER pediatric intervals (Bohn et al., 2023) (15). Immunoglobulin and lymphocyte subset ranges were based on the PID Phenotypical Diagnosis App (Jeddane et al., 2017) and Shearer et al., 2003;112:973–80 (16, 17). WBC, white blood cells; NK, natural killer cells. Symbols: ↑, above reference range; ↓, below reference range. ACMG: American College of Medical Genetics and Genomics variant classification system (PVS1 = very strong pathogenic evidence; PM2 = moderate; PS4 = Pathogenic Strong; PP5= Pathogenic Supporting; PP3= Pathogenic Supporting).

Family history revealed a brother with a similar clinical presentation including chronic immune thrombocytopenic purpura and hypogammaglobulinemia who received similar treatment and also died from severe bleeding complicated by shock. The similarity of clinical and laboratory features strongly suggests a shared underlying etiology consistent with a primary immunodeficiency, specifically LRBA deficiency (**Table 2**).

**Table 2:** Clinical characteristics on the five Moroccan patients with LRBA deficiency.

Characteristics	Case 1	Case 2	Case 3	Case 4	Case 5
Age at onset of clinical signs	11 years	7 months	6 months	2 years	4 years
Age at diagnosis	15 years	2 years	3 years	5 years	16 years
Sex	Female	Female	Female	Female	Female
Consanguinity	Yes (second-degree)	Yes (second-degree)	Yes (first-degree)	Yes (first-degree)	Yes (first-degree)
Clinical manifestations	Growth retardation, mild intellectual disability, hypogammaglobulinemia, recurrent episodes of cutaneous purpura	Hemorrhagic syndrome, anemic syndrome	Pica, febrile diarrhea, pallor of the skin and mucous membranes, fever, respiratory distress	Fever, growth retardation, diffuse abdominal distension, chronic diarrhea, weight loss, vomiting, arthritis, respiratory involvement	Chronic diarrhea, abdominal pain, petechial purpura, growth retardation, bronchitis, chronic cough, lymph node tuberculosis, bilateral panuveitis, arthritis, agammaglobulinemia
Lymphoproliferation	None	Splenomegaly and hepatomegaly	Splenomegaly	Splenomegaly, hepatomegaly and Lymphadenopathy	None
Autoimmune manifestations	ITP and AIHA	ITP	AIHA	Bicytopenia (AIHA and autoimmune neutropenia), type 1 diabetes, enteropathy	ITP
Family history	A brother deceased in a similar context	History of three deaths within the sibling group	No similar cases in the family	Death of a brother at 8 months of age	No similar cases in the family

#### Family 2 (case 2)

**Patient 2** is a 2-year-old girl (**Figure 1A**), the youngest of nine siblings, born to a second-degree consanguineous marriage. She has been followed since the age of 7 months for a progressive hemorrhagic syndrome associated with cutaneous and mucosal pallor, related to immune thrombocytopenic purpura. Clinical examination revealed splenomegaly and hepatomegaly. There was no history of recurrent infections at the onset of clinical manifestations.

The complete blood count revealed severe bicytopenia, with profound anemia (hemoglobin: 5.9 g/dL) and severe thrombocytopenia (platelets: 4,000/mm³), in the context of moderate leukopenia (white blood cells: 4,080/mm³), associated with marked neutropenia (neutrophils: 890/mm³) and lymphocytes at 1,013/mm³. Despite treatment with corticosteroids and cyclosporine, thrombocytopenia persisted at 20,000/mm³. Bone marrow examination suggested congenital dyserythropoiesis type 2 or 5. Lymphocyte immunophenotyping showed T and B lymphopenia with reduced total T cells CD3 (1,569/mm³), decreased CD4+ T cells (697/mm³), normal CD8+ T cells (717/mm³), decreased B cells CD19 (58/mm³), and preserved NK cells (252/mm³). Serum immunoglobulin levels were within normal ranges for age with IgG 4.88 g/L, IgA 0.68 g/L, IgM 0.95 g/L, and IgE 13.90 IU/mL (**Table 1**). The patient required repeated transfusions of red blood cells and platelets due to the severity of anemia and thrombocytopenia. Initial immunosuppressive therapy consisting of full-dose corticosteroids combined with cyclosporine (Neoral^®^) at 5 mg/kg/day, administered in two divided doses, was initiated. However, significant clinical and laboratory improvement was observed only after the introduction of abatacept, leading to regression of splenomegaly and gradual normalization of hematologic parameters.

Family history revealed three sibling deaths, one at 12 years from severe hemorrhagic syndrome, one at 11 months associated with malnutrition and chronic diarrhea, and one at 1.5 months due to respiratory distress. These familial factors, combined with parental consanguinity and the patient’s clinical presentation, strongly suggest an underlying primary immunodeficiency, consistent with LRBA deficiency (**Table 2**).

#### Family 3 (case 3)

Patient 3 is a 3-year-old girl, an only child, born from a first-degree consanguineous marriage (**Figure 1A**). Her medical history is notable for recurrent episodes of transfusion-refractory hemolytic anemia, associated with pica, febrile diarrhea, and prolonged fever. Clinical examination revealed massive splenomegaly. In the presence of a positive direct Coombs test, a diagnosis of autoimmune hemolytic anemia was established. During follow-up, the clinical course was complicated by hemoptysis. Chest radiography revealed persistent pneumonia despite appropriate antibiotic therapy, leading to the initiation of antibacillary treatment.

Complete blood count showed moderate anemia (hemoglobin 9.8 g/dL), leukopenia (2,010/mm³) with severe neutropenia (310/mm³) and lymphopenia (1,150/mm³), relatively preserved platelet count (141,000/mm³). Lymphocyte immunophenotyping revealed combined T, B, and NK cell lymphopenia with decreased total T cells (CD3^+^ 417/mm³), CD4^+^ T cells (206/mm³), CD8^+^ T cells (183/mm³), decreased B cells (CD19^+^ 190/mm³), and decreased NK cells (63/mm³). Serum immunoglobulin levels were within normal ranges with IgG 5.97 g/L, IgA 2.48 g/L, IgM 0.54 g/L, and IgE 50 IU/mL (**Table 1**).

Therapeutic management included intravenous immunoglobulin administration and prophylaxis with trimethoprim-sulfamethoxazole. For the treatment of autoimmune hemolytic anemia, corticosteroids combined with cyclosporine and mycophenolate mofetil (CellCept^®^) were initiated, without significant clinical or laboratory improvement. The clinical course was complicated by recurrent infections and inflammatory colitis, prompting the introduction of abatacept. This treatment led to improvement of the colitis, while pulmonary involvement persisted. Chest computed tomography (CT) revealed interstitial lung disease without bronchial dilation. Blood PCR for cytomegalovirus (CMV) was negative. A bronchoalveolar lavage was performed, which showed no evidence of Pneumocystis jirovecii.

#### Family 4 (case 4)

Patient 4 is a 5-year-old girl, born from a first-degree consanguineous marriage. Family history revealed the death of a brother at 8 months of age in the context of febrile coma (**Figure 1A**).

She was first hospitalized at 2 years of age for prolonged fever. Clinically, she presented with chronic diarrhea, abdominal pain, vomiting, and growth retardation. Examination revealed hepatomegaly and splenomegaly, and abdominal ultrasound showed lymphadenopathy. Based on these findings and radiological abnormalities, intestinal tuberculosis was suspected, and anti-tuberculosis therapy was initiated. The clinical course was complicated by hepatic cytolysis. Subsequently, the patient developed arthritis of the left hip, initially treated as infectious despite sterile joint fluid cultures. Reevaluation of imaging revealed osteolytic lesions involving the pelvis and distal ends of both femurs, confirmed on bone-window computed tomography. She also experienced recurrent episodes of hypocalcemia secondary to enteropathy with vitamin D deficiency, requiring repeated hospitalizations for electrolyte correction. An interstitial lung disease later developed, manifested by chest pain and persistent cough, in the context of general deterioration with asthenia, anorexia, and feeding refusal. Finally, the patient developed type 1 diabetes mellitus, revealed by an inaugural episode of diabetic ketoacidosis.

Biological evaluation revealed bicytopenia, with severe anemia (hemoglobin 5.8 g/dL) and neutropenia (1,100/mm³), associated with leukopenia (1,980/mm³), lymphopenia (770/mm³), and a relatively preserved platelet count (180,000/mm³). Lymphocyte immunophenotyping demonstrated decreased subpopulations: CD4 T cells (325/mm³), CD19 B cells (234/mm³), and NK cells (103/mm³), whereas CD3 T cells (2,213/mm³) and CD8 T cells (503/mm³) counts were relatively preserved. Serum immunoglobulin levels were within the normal range for age: IgG 7.54 g/L, IgA 0.42 g/L, and IgM 1.01 g/L (**Table 1**). Initial management included systemic corticosteroids combined with anti-tuberculosis therapy, along with monthly intravenous immunoglobulin replacement. Despite this treatment, clinical and biological evolution remained unfavorable with no significant improvement. Following the introduction of abatacept at the age of 4 years, a marked clinical improvement was observed with favorable hematological, digestive, and skeletal responses. However, the patient subsequently died from respiratory distress.

#### Family 5 (case 5)

Patient 5 is a 16-year-old girl born from a first-degree consanguineous marriage (Figur 1A). Her clinical history began at the age of 4 years with chronic diarrhea associated with recurrent abdominal pain. She was subsequently hospitalized for petechial and ecchymotic purpura preceded by episodes of severe epistaxis, leading to the diagnosis of immune thrombocytopenic purpura. Growth retardation was noted at the age of 10 years. At 11 years old, she developed a suspected lymph node tuberculosis, which was treated with a complete anti-tuberculosis regimen. From the age of 13 years, her clinical course was marked by recurrent lower respiratory tract infections, including frequent episodes of bronchitis, chronic productive cough with greenish sputum, and recurrent otitis media complicated by purulent otorrhea, occurring at a frequency of approximately four episodes per year. The disease course was further complicated by severe ocular involvement characterized by bilateral panuveitis associated with vitreous and retinal hemorrhages in the context of vasculitis. In addition, rheumatologic manifestations were observed, including right knee arthritis and inflammatory peripheral arthralgia.

Complete blood count revealed moderate anemia (hemoglobin 9.9 g/dL), associated with leukocytosis (14,300/mm³) with neutrophil predominance (12,870/mm³), marked lymphopenia (715/mm³), and moderate thrombocytopenia (126,000/mm³). Lymphocyte immunophenotyping showed decreased CD4 T cells (455/mm³) and absent CD19 B cells (0/mm³), whereas CD3 T cells (1,125/mm³), CD8 (638/mm³), and NK cells (89/mm³) were within normal limits. Serum immunoglobulin quantification demonstrated agammaglobulinemia, with undetectable IgG (0.00 g/L), markedly reduced IgM (0.1 g/L), and decreased IgA (0.24 g/L) (**Table 1**).

Several courses of corticosteroid therapy resulted in transient clinical improvement, followed by worsening of inflammatory colitis. Immunoglobulin replacement therapy was initiated. For ocular involvement, immunosuppressive treatment combining azathioprine (Imurel^®^ 100 mg/day) and prednisone (Cortancyl^®^ 40 mg/day) was introduced. The introduction of abatacept led to marked clinical improvement.

### Genetic findings

Genetic analysis identified homozygous pathogenic variants in the LRBA gene (NM_006726.5) in all five patients, all of which are consistent with a loss-of-function mechanism.

In Patient 1 (Case 1), the variant c.3811C>T (p.Arg1271*) was identified. This nonsense variant introduces a premature termination codon and is therefore predicted to result in loss of function through nonsense-mediated mRNA decay (PVS1). The variant is extremely rare in population databases, with an allele frequency of 0.00000137 (2/1,455,696 chromosomes) in gnomAD and no reported homozygous individuals (PM2). In silico predictions support a deleterious effect (PP3), and the variant is classified as pathogenic in ClinVar (PP5). Based on these criteria (PVS1, PM2, PP3, PP5), the variant was classified as pathogenic.

Patient 2 (Case 2) carried the variant c.5692G>T (p.Glu1898*), a nonsense mutation affecting a conserved nucleotide and introducing a premature stop codon. This variant is expected to cause loss of function via nonsense-mediated mRNA decay (PVS1). It is absent from population databases, including gnomAD (PM2), and is reported as pathogenic in ClinVar (PP5). Taken together (PVS1, PM2, PP5), this variant was classified as pathogenic.

In Patient 3 (Case 3), a large homozygous deletion encompassing exons 30 to 34 of the LRBA gene was identified. This copy number variation results in an out-of-frame transcript with a predicted premature stop codon, leading to absent or severely truncated protein (PVS1). Loss-of-function variants in LRBA are a well-established cause of disease (PVS1_strong). Similar deletions have been reported in patients with primary immunodeficiency phenotypes (PS4_supporting). As the variant is absent from population databases (PM2), it was classified as pathogenic according to ACMG criteria (PVS1, PM2, PS4_supporting).

Patient 4 (Case 4) was found to carry the variant c.5060_5067delACATACCA (p.Asn1687Serfs*21), a frameshift deletion leading to a premature stop codon. This variant is predicted to result in loss of function through nonsense-mediated mRNA decay (PVS1). It is absent from population databases such as gnomAD (PM2) and is classified as pathogenic in ClinVar (PP5). According to ACMG criteria (PVS1, PM2, PP5), this variant was classified as pathogenic.

Finally, in Patient 5 (Case 5), a homozygous deletion involving part of exon 4 of the LRBA gene (c.476_549 + 580del) was identified. This alteration is predicted to disrupt normal mRNA splicing and is therefore expected to result in loss of function (PVS1). The variant is absent from population databases (PM2). As no direct functional studies or multiple independent reports were available, the evidence supports a classification of likely pathogenic according to ACMG criteria (PVS1, PM2).

## Discussion

The LRBA protein plays a crucial role in maintaining immune homeostasis by regulating the expression and intracellular recycling of CTLA-4, a major inhibitory molecule expressed by activated T lymphocytes and Tregs. CTLA-4 limits T-cell co-stimulation and proliferation, thereby contributing to the control of peripheral immune responses (18, 19). In the context of LRBA deficiency, reduced CTLA-4 expression, together with decreased FoxP3 and CD25 levels, impairs the suppressive function of Tregs. This dysregulation leads to a partial loss of control over T-cell activation, resulting in impaired activation of both T and B lymphocytes. Consequently, defective immune tolerance and immune surveillance promote the development of autoimmune manifestations, lymphoproliferative disorders, and increase the risk of malignancy (20, 21). In this study, we report five patients from five distinct Moroccan families, each harboring a pathogenic homozygous mutation in the *LRBA* gene. These observations illustrate the major phenotypic features of LRBA deficiency and highlight the importance of this condition in the differential diagnosis of immunodeficiency disorders associated with immune dysregulation. Moreover, our findings provide valuable insights to optimize diagnostic strategies and therapeutic management within our population context.

Patients with LRBA deficiency exhibit a particularly heterogeneous clinical and immunological spectrum. Immune dysregulation represents the predominant clinical hallmark of this condition, as more than 90% of patients develop at least one autoimmune manifestation during the course of the disease (9, 19, 22). The most frequently reported autoimmune manifestations are autoimmune cytopenias, particularly AIHA and ITP. Chronic diarrhea, often associated with a poor therapeutic response, contributes significantly to growth failure, which is observed in approximately 25% of patients with LRBA deficiency (3, 8, 20, 22). Other autoimmune and inflammatory manifestations may also occur, albeit less frequently, including juvenile idiopathic arthritis, neutropenia, chronic autoimmune hepatitis, eczema, type 1 diabetes mellitus, autoimmune thyroiditis, arthritis, uveitis, psoriasis, vitiligo, and alopecia, highlighting the marked phenotypic variability of this disorder (14, 23, 24). Our findings are consistent with previously published data reporting a high prevalence of autoimmune cytopenias in patients with LRBA deficiency. In our cohort, these manifestations represented the most commonly observed autoimmune features. Patient 1 presented with both AIHA and ITP, Patient 2 with isolated ITP, whereas Patient 3 developed AIHA. Patient 4 exhibited a more severe and multisystemic phenotype, characterized by autoimmune bicytopenia (AIHA and autoimmune neutropenia), type 1 diabetes mellitus, and enteropathy, corresponding to a fully expressed clinical presentation of LRBA deficiency. Patient 5 presented with ITP.

In addition, manifestations of organomegaly and lymphoproliferation are frequently reported in patients with LRBA deficiency, with an estimated prevalence ranging from 76% to 86%. The most commonly observed features include splenomegaly, peripheral lymphadenopathy, and hepatomegaly (14, 23, 24), reflecting chronic immune activation and dysregulation of lymphocyte proliferation control. In line with data from the literature reporting a high prevalence of lymphoproliferative manifestations and organomegaly in LRBA deficiency, our clinical observations confirm this trend. In our cohort, Patient 1 did not exhibit any documented lymphoproliferative manifestations or organomegaly. In contrast, splenomegaly associated with hepatomegaly was observed in Patient 2, whereas Patient 3 presented with isolated splenomegaly. Patient 4 showed a combination of splenomegaly, hepatomegaly, and lymphadenopathy. Hypogammaglobulinemia is a frequent finding in LRBA deficiency, affecting approximately 50–80% of patients (11). In our series, this abnormality was observed in Patients 1 and 5. This impairment of humoral immunity highlights the need for comprehensive immunological evaluation and regular follow-up, particularly in view of the variability in vaccine responses reported in this condition. Regarding neurological involvement, complications have been described in approximately 20% of patients with LRBA deficiency (14, 23). In our cohort, neurological involvement was identified only in Patient 1, who presented with intellectual disability, while no neurological manifestations were observed in the remaining patients. This finding further illustrates the marked phenotypic heterogeneity of LRBA deficiency and the variability of associated neurological involvement. Consanguinity was observed in all patients in our cohort. LRBA deficiency follows an autosomal recessive mode of inheritance, and consanguinity significantly increases the risk of homozygosity for rare pathogenic variants. In populations where consanguineous marriages are common, as in our setting, this practice represents a major contributing factor to the emergence and clinical expression of IEIs, particularly those related to immune regulation defects such as LRBA deficiency. The high rate of consanguinity in our series underscores the need for a high index of clinical suspicion. Moreover, these findings reinforce the importance of family genetic counseling, which is essential for identifying at-risk relatives and preventing the occurrence of new cases within affected families.

From a genetic perspective, all variants identified in our patients correspond to alterations leading to a loss-of-function of the *LRBA* gene. In Case 1, the nonsense variant c.3811C>T (p.Arg1271*) introduces a premature stop codon; it is extremely rare in the general population, absent in the homozygous state in gnomAD, predicted to be deleterious by in silico analyses, and classified as pathogenic in ClinVar, with an expected mechanism of mRNA degradation through nonsense-mediated decay (NMD). Similarly, the patient in Case 2 carried the nonsense variant c.5692G>T (p.Glu1898*), which is absent from population databases and classified as pathogenic in ClinVar, and is also likely to induce NMD-mediated mRNA degradation. In Case 3, a large deletion encompassing exons 30 to 34 of the *LRBA* gene was identified, resulting in a frameshift and the introduction of a premature stop codon, leading to absence or severe truncation of the protein. This type of loss-of-function variant is well established as pathogenic in the literature. The patient in Case 4 harbored the deletion c.5060_5067delACATACCA (p.Asn1687Serfs*21), which is absent from population databases, classified as pathogenic in ClinVar, and associated with an expected NMD-mediated mRNA degradation mechanism. Overall, these findings confirm that the phenotypes observed in our series are associated with severe genetic alterations of *LRBA*, in agreement with previously published data. Finally, in Patient 5, the deletion c.476_549 + 580del affecting part of exon 4 of the *LRBA* gene is highly suggestive of a pathogenic mechanism involving splicing disruption, potentially resulting in a truncated or non-functional protein. Splice-altering variants are well recognized to cause loss of function, a central mechanism in the pathophysiology of LRBA deficiency. Although the absence of functional data limits definitive interpretation, the convergence of genetic and clinical evidence supports a causal role of this variant in the observed phenotype. Nevertheless, additional functional studies would be required to formally confirm its biological impact and direct involvement in disease pathogenesis.

Although the precise biological role of LRBA has not yet been fully elucidated, accumulating evidence indicates that this protein is essential for the post-translational expression and intracellular trafficking of CTLA-4 (20, 22). CTLA-4 is an inhibitory receptor expressed on the surface of activated conventional T lymphocytes as well as Tregs. Upon T-cell receptor (TCR) stimulation, CTLA-4 is mobilized from intracellular vesicular compartments to the cell membrane, where it captures the co-stimulatory molecules CD80 and CD86 expressed by antigen-presenting cells (APCs) through a transendocytosis mechanism, thereby negatively regulating pro-inflammatory immune responses (4). Several negative regulatory mechanisms controlling CTLA-4 expression have been identified, involving proteins that modulate its transcriptional and post-translational regulation. Among these, the adaptor protein complex AP-1 binds to the YVKM tetrapeptide motif within the cytoplasmic tail of CTLA-4, promoting its targeting to AP-1–containing vesicles and subsequent lysosomal degradation (22). In contrast, LRBA facilitates the recycling of CTLA-4–containing vesicles back to the Treg cell surface by binding to the same YVKM motif, thereby preventing AP-1 interaction and limiting lysosomal degradation of CTLA-4 (20).

In the absence of LRBA, as observed in patients with LRBA deficiency, increased lysosomal degradation of CTLA-4 occurs, leading to a marked reduction in its protein levels. This decreased CTLA-4 expression impairs the suppressive function of Tregs and contributes to defective regulation of immune responses, thereby promoting the development of autoimmune manifestations and lymphoproliferative syndromes, which are frequently described in this condition (18, 20). In addition, B-cell abnormalities and antibody deficiency observed in patients with LRBA deficiency appear to be linked to increased apoptosis of B lymphocytes secondary to defective autophagy, particularly under conditions of cellular stress or serum deprivation (6). It is well established that both short- and long-lived plasma cells must remodel and expand their endoplasmic reticulum to sustain increased immunoglobulin production following antigenic stimulation, a process that generates substantial oxidative, proteasomal, and endoplasmic reticulum stress. Under physiological conditions, this stress is compensated by enhanced autophagy, which supports cellular survival and energy production. In contrast, impaired autophagy results in reduced B-cell survival and decreased intracellular ATP levels (25). These data underscore the central role of autophagy in plasma cell differentiation and suggest that autophagy defects related to LRBA deficiency may account for the reduced numbers of switched memory B cells and plasmablasts observed in patients with LRBA deficiency.

The management of LRBA deficiency is primarily focused on controlling hypogammaglobulinemia, infections, lymphoproliferation, and autoimmune manifestations. Hypogammaglobulinemia is treated with immunoglobulin replacement therapy, administered either intravenously or subcutaneously, and combined with appropriate antimicrobial treatment in the presence of infections (11, 14, 22). Autoimmune and lymphoproliferative manifestations frequently require immunosuppressive therapy, including corticosteroids, rituximab, hydroxychloroquine, mycophenolate mofetil, or azathioprine. However, these conventional treatments do not always prevent long-term disease progression. Sirolimus has shown particular efficacy in the control of enteropathy, with improvement of chronic diarrhea and subsequent weight gain reported in several patients (22, 26, 27). Given the central role of CTLA-4 in the pathophysiology of LRBA deficiency, abatacept has emerged as a targeted therapeutic option. Long-term follow-up studies have demonstrated overall clinical improvement, including enhanced pulmonary function, accompanied by reduced T cell–mediated inflammation and improved antigen-specific immune responses (19).

Finally, hematopoietic stem cell transplantation (HSCT) represents a potentially curative therapeutic option and is currently recommended, when a suitable donor is available, prior to the development of irreversible organ damage that may compromise its efficacy (22).

This study has several limitations. First, the small sample size and the descriptive nature of this case series limit the generalizability of the findings and do not allow the establishment of robust causal relationships. Second, the marked phenotypic variability of LRBA deficiency, together with the heterogeneity of clinical presentations and therapeutic approaches, complicates systematic comparisons between patients. Third, some advanced immunological investigations, including Treg evaluation, detailed B-cell subset analysis, and functional CTLA-4 assays, were not available for our patients, which may limit the mechanistic interpretation of the results. Despite the small cohort size and limited immunological characterization, this series offers complementary observations that contribute to the existing literature on LRBA deficiency in the Moroccan population.

## Conclusion

LRBA deficiency is a rare inborn error of immunity characterized by marked clinical and immunological heterogeneity, predominantly driven by immune dysregulation, autoimmune cytopenias, and lymphoproliferative manifestations. Through this series of five patients from distinct Moroccan families, we highlight the wide spectrum of clinical presentations, ranging from limited phenotypes to severe multisystemic disease, underscoring the diagnostic challenges associated with this condition. Our observations confirm the central role of the LRBA–CTLA-4 pathway in maintaining immune tolerance and illustrate the therapeutic value of targeted treatments, particularly abatacept and sirolimus, in the management of severe or refractory forms. Early recognition of LRBA deficiency especially in patients presenting with early-onset autoimmunity, recurrent cytopenias, and/or hypogammaglobulinemia in the context of consanguinity is essential to optimize patient management and prevent long-term complications. Multicenter studies involving larger cohorts and extended follow-up are needed to better define prognostic factors, standardize therapeutic strategies, and clarify the role of hematopoietic stem cell transplantation in the management algorithm of LRBA deficiency.

## Data Availability

Datasets are available on request: The raw data supporting the conclusions of this article will be made available by the authors, without undue reservation.

## References

[1] Habibi S Zaki-Dizaji M Rafiemanesh H Lo B Jamee M Gámez-Díaz L . Clinical, immunologic, and molecular spectrum of patients with LPS-responsive beige-like anchor protein deficiency: a systematic review. J Allergy Clin Immunology: In Pract. (2019) 7:2379–2386.e5. doi: 10.1016/j.jaip.2019.04.011. PMID: 30995531

[2] Burnett DL Parish IA Masle-Farquhar E Brink R Goodnow CC . Murine LRBA deficiency causes CTLA-4 deficiency in Tregs without progression to immune dysregulation. Immunol Cell Biol. (2017) 95:775–88. doi: 10.1038/icb.2017.50. PMID: 28611475 PMC5636941

[3] Meshaal S El Hawary R Adel R Abd Elaziz D Erfan A Lotfy S . Clinical phenotypes and immunological characteristics of 18 Egyptian LRBA deficiency patients. J ClinImmunol. (2020) 40:820–32. doi: 10.1007/s10875-020-00799-2. PMID: 32506362

[4] Qureshi OS Zheng Y Nakamura K Attridge K Manzotti C Schmidt EM . Trans-endocytosis of CD80 and CD86: a molecular basis for the cell-extrinsic function of CTLA-4. Science. (2011) 332:600–3. doi: 10.1126/science.1202947. PMID: 21474713 PMC3198051

[5] Charbonnier LM Janssen E Chou J Ohsumi TK Keles S Hsu JT . Regulatory T-cell deficiency and immune dysregulation, polyendocrinopathy, enteropathy, X-linked–like disorder caused by loss-of-function mutations in LRBA. J Allergy Clin Immunol. (2015) 135:217–227.e9. doi: 10.1016/j.jaci.2014.10.019. PMID: 25468195 PMC4289093

[6] Lopez-Herrera G Tampella G Pan-Hammarström Q Herholz P Trujillo-Vargas CM Phadwal K . Deleterious mutations in LRBA are associated with a syndrome of immune deficiency and autoimmunity. Am J Hum Genet. (2012) 90:986–1001. doi: 10.1016/j.ajhg.2012.04.015. PMID: 22608502 PMC3370280

[7] Bousfiha AA Jeddane L Moundir A Poli MC Aksentijevich I Cunningham-Rundles C . The 2024 update of IUIS phenotypic classification of human inborn errors of immunity. J Hum Immun. (2025) 1:e20250002. doi: 10.70962/jhi.20250002. PMID: 41608113 PMC12829316

[8] Alangari A Alsultan A Adly N Massaad MJ Kiani IS Aljebreen A . LPS-responsive beige-like anchor (LRBA) gene mutation in a family with inflammatory bowel disease and combined immunodeficiency. J Allergy Clin Immunol. (2012) 130:481–488.e2. doi: 10.1016/j.jaci.2012.05.043. PMID: 22721650 PMC3582381

[9] Kiykim A Ogulur I Dursun E Charbonnier LM Nain E Cekic S . Abatacept as a long-term targeted therapy for LRBA deficiency. J Allergy Clin Immunol Pract. (2019) 7:2790–2800.e15. doi: 10.1016/j.jaip.2019.06.011. PMID: 31238161 PMC6842687

[10] Alroqi FJ Charbonnier LM Baris S Kiykim A Chou J Platt CD . Exaggerated follicular helper T-cell responses in patients with LRBA deficiency caused by failure of CTLA4-mediated regulation. J Allergy Clin Immunol. (2018) 141:1050–1059.e10. doi: 10.1016/j.jaci.2017.05.022. PMID: 28601686 PMC5743769

[11] Gámez-Díaz L August D Stepensky P Revel-Vilk S Seidel MG Noriko M . The extended phenotype of LPS-responsive beige-like anchor protein (LRBA) deficiency. J Allergy Clin Immunol. (2016) 137:223–30. doi: 10.1016/j.jaci.2015.09.025, PMID: 26768763

[12] Jiang L Chen S . Case report: A case of novel homozygous LRBA variant induced by chromosomal segmental uniparental disomy - genetic and clinical insights. Front Immunol. (2024) 15. doi: 10.3389/fimmu.2024.1351076, PMID: 38504982 PMC10948553

[13] Perez-Perez D Santos-Argumedo L Rodriguez-Alba JC Lopez-Herrera G . Analysis of LRBA pathogenic variants and the association with functional protein domains and clinical presentation. Pediatr Allergy Immunol. (2024) 35:e14179. doi: 10.1111/pai.14179. PMID: 38923448

[14] Alkhairy OK Abolhassani H Rezaei N Fang M Andersen KK Chavoshzadeh Z . Spectrum of phenotypes associated with mutations in LRBA. J ClinImmunol. (2016) 36:33–45. doi: 10.1007/s10875-015-0224-7. PMID: 26707784

[15] Bohn MK Wilson S Steele S Adeli K . Comprehensive pediatric reference intervals for 79 hematology markers in the CALIPER cohort of healthy children and adolescents using the Mindray BC-6800Plus system. Int J Lab Hematol. (2023) 45:469–80. doi: 10.1111/ijlh.14068. PMID: 36990763

[16] Bayram RO Özdemir H Emsen A Türk Daği H Artaç H . Reference ranges for serum immunoglobulin (IgG, IgA, and IgM) and IgG subclass levels in healthy children. Turk J Med Sci. (2019) 49:497–505. doi: 10.3906/sag-1807-282. PMID: 30997788 PMC7018341

[17] Shearer WT Rosenblatt HM Gelman RS Oyomopito R Plaeger S Stiehm ER . Lymphocyte subsets in healthy children from birth through 18 years of age: the Pediatric AIDS Clinical Trials Group P1009 study. J Allergy Clin Immunol. (2003) 112:973–80. doi: 10.1016/j.jaci.2003.07.003. PMID: 14610491

[18] Lo B Fritz JM Su HC Uzel G Jordan MB Lenardo MJ . CHAI and LATAIE: new genetic diseases of CTLA-4 checkpoint insufficiency. Blood. (2016) 128:1037–42. doi: 10.1182/blood-2016-04-712612. PMID: 27418640 PMC5000841

[19] Cagdas D Halaçlı SO Tan Ç Lo B Çetinkaya PG Esenboğa S . A spectrum of clinical findings from ALPS to CVID: several novel LRBA defects. J ClinImmunol. (2019) 39:726–38. doi: 10.1007/s10875-019-00677-6. PMID: 31432443 PMC11090043

[20] Lo B Zhang K Lu W Zheng L Zhang Q Kanellopoulou C . Autoimmune disease. Patients with LRBA deficiency show CTLA4 loss and immune dysregulation responsive to abatacept therapy. Science. (2015) 349:436–40. doi: 10.1126/science.aaa1663. PMID: 26206937

[21] Riaz IB Faridi W Patnaik MM Abraham RS . A systematic review on predisposition to lymphoid (B and T cell) neoplasias in patients with primary immunodeficiencies and immune dysregulatory disorders (inborn errors of immunity). Front Immunol. (2019) 10:777. doi: 10.3389/fimmu.2019.00777. PMID: 31057537 PMC6477084

[22] Gámez-Díaz L . LRBA deficiency. In: D’Elios MM Rizzi M , editors.Humoral Primary Immunodeficiencies. Springer International Publishing, Cham (2019). p. 113–29. doi: 10.1007/978-3-319-91785-6_10, PMID:

[23] Azizi G Abolhassani H Mahdaviani SA Chavoshzadeh Z Eshghi P Yazdani R . Clinical, immunologic, molecular analyses and outcomes of Iranian patients with LRBA deficiency: a longitudinal study. Pediatr Allergy Immunol. (2017) 28:478–84. doi: 10.1111/pai.12735. PMID: 28512785

[24] Kostel Bal S Haskologlu S Serwas NK Islamoglu C Aytekin C Kendirli T . Multiple presentations of LRBA deficiency: a single-center experience. J ClinImmunol. (2017) 37:790–800. doi: 10.1007/s10875-017-0446-y. PMID: 28956255 PMC7086713

[25] Pengo N Scolari M Oliva L Milan E Mainoldi F Raimondi A . Plasma cells require autophagy for sustainable immunoglobulin production. Nat Immunol. (2013) 14:298–305. doi: 10.1038/ni.2524. PMID: 23354484

[26] Azizi G Abolhassani H Yazdani R Mohammadikhajehdehi S Parvaneh N Negahdari B . New therapeutic approach by sirolimus for enteropathy treatment in patients with LRBA deficiency. Eur Ann Allergy Clin Immunol. (2017) 49:235–9. doi: 10.23822/eurannaci.1764-1489.22. PMID: 28884992

[27] Seidel MG Hirschmugl T Gamez-Diaz L Schwinger W Serwas N Deutschmann A . Long-term remission after allogeneic hematopoietic stem cell transplantation in LPS-responsive beige-like anchor (LRBA) deficiency. J Allergy Clin Immunol. (2015) 135:1384–1390.e1-8. doi: 10.1016/j.jaci.2014.10.048. PMID: 25539626 PMC4429722

